# Evaluating past and future contributions of conservation programs to species recovery

**DOI:** 10.1111/cobi.70183

**Published:** 2025-11-25

**Authors:** Rebeca E. Young, H. Resit Akçakaya, Elizabeth L. Bennett, Michael Hoffmann, Michael A. Hudson, Barney Long, Thalassa McMurdo Hamilton, Kelsey Neam, Megan A. Owen, Richard P. Young, Molly K. Grace

**Affiliations:** ^1^ Durrell Wildlife Conservation Trust Trinity UK; ^2^ Department of Ecology and Evolution Stony Brook University Stony Brook New York USA; ^3^ Wildlife Conservation Society Bronx New York USA; ^4^ Zoological Society of London London UK; ^5^ Re:wild Washington, DC USA; ^6^ San Diego Zoo Wildlife Alliance San Diego California USA; ^7^ Nature Positive Bath UK; ^8^ Department of Biology University of Oxford Oxford UK

**Keywords:** conservation impact, impact evaluation, IUCN, IUCN Green Status of Species, program, program evaluation, red list, species recovery, Estado Verde de las Especies de la UICN, evaluación de impactos, evaluación de programas, impacto de la conservación, lista roja, programa, recuperación de especies, UICN, 影响评估, 保护影响, 物种恢复, 项目, 世界自然保护联盟(IUCN), 世界自然保护联盟物种绿色状况, 红色名录, 项目评估

## Abstract

Impact evaluation of conservation actions is a crucial step in global efforts to curb the biodiversity crisis. Through robust impact evaluation, practitioners can assess the effectiveness of conservation strategies and optimize the use of limited resources. Despite a proliferation of methods and tools for evaluating conservation impact, no standardized method exists to assess and compare the impact, and global contribution, of species recovery programs. To address this gap, we devised an evaluation framework, based on the International Union for Conservation of Nature (IUCN) Green Status of Species (GSS), a global standard for measuring species recovery. We sought to provide a way for conservation program delivery partners to evaluate the effectiveness of their programs in contributing to global species recovery. We adapted 2 scenarios used in GSS assessments to estimate the impact of worldwide conservation actions on a species (the counterfactual current scenario and the future without conservation scenario), in order to propose a new assessment: the program GSS, a method allowing conservation practitioners to estimate the past and potential future impacts of a conservation program relative to the global impact. To identify the strengths and limitations of applying the GSS method at the program level and to gather proof of concept for our adaptation, we tested the proposed method on 16 species recovery programs. The program GSS approach identified past or future impacts of program actions on species status in 9 of the programs assessed. The detectability of program impact and the relative impact of the program compared with global impact were influenced by time since program establishment and program scope (i.e., proportion of a species’ population or distribution included in the program). Our framework for program GSS assessments can provide practitioners with a standard, straightforward, and cost‐effective way to communicate conservation successes and expected future impacts. Results from our program GSS framework can be compared with the global recovery of a species (conservation legacy and conservation impact) and thus indicate a program's contribution to the recovery of the entire species.

## Abstract (es)

Evaluación de las contribuciones previas y futuras de los programas de conservación a la recuperación de las especies

**Resumen:**

La evaluación del impacto de las medidas de conservación es un paso crucial en los esfuerzos mundiales para frenar la crisis de la biodiversidad. Con una evaluación de impacto sólida, los profesionales pueden evaluar la eficacia de las estrategias de conservación y optimizar el uso de los recursos limitados. A pesar de los muchos métodos y herramientas para evaluar el impacto de la conservación, no existe un método estandarizado para evaluar y comparar el impacto y la contribución global de los programas de recuperación de especies. Para llenar este vacío, diseñamos un marco de evaluación basado en el Estado Verde de las Especies (GSS, por sus siglas en inglés) de la Unión Internacional para la Conservación de la Naturaleza, un estándar global para medir la recuperación de especies. Nuestro objetivo fue proporcionar a los socios encargados de la ejecución de programas de conservación una forma de evaluar la eficacia de sus programas en la contribución a la recuperación global de las especies. Adaptamos dos escenarios utilizados en las evaluaciones del GSS para estimar el impacto de las acciones de conservación a nivel mundial sobre una especie (el escenario actual contrafactual y el escenario futuro sin conservación), con el fin de proponer una nueva evaluación: el programa GSS, un método que permite que los profesionales de la conservación estimen los impactos pasados y futuros potenciales de un programa de conservación en relación con el impacto global. Para identificar las fortalezas y limitaciones de la aplicación del método GSS a nivel de programa y recopilar pruebas de concepto para nuestra adaptación, probamos el método propuesto en 16 programas de recuperación de especies. El enfoque GSS del programa identificó los impactos pasados y futuros de las acciones del programa sobre el estado de las especies en 7 de los programas evaluados. La detectabilidad del impacto del programa y el impacto relativo del programa en comparación con el impacto global dependían del tiempo transcurrido desde el establecimiento del programa y de su alcance (es decir, la proporción de la población o distribución de una especie incluida en el programa). Nuestro marco para las evaluaciones del GSS de los programas puede proporcionar a los profesionales una forma estándar, sencilla y rentable de comunicar los éxitos en materia de conservación y los impactos futuros previstos. Los resultados de nuestro marco GSS de programas pueden compararse con la recuperación global de una especie (legado de conservación e impacto de la conservación) y, por lo tanto, indicar la contribución de un programa a la recuperación de toda la especie.

## 评估保护项目过去和未来对物种恢复的贡献

**【摘要】** 对保护行动进行影响评估是全球遏制生物多样性危机工作的关键步骤之一。保护工作者可以通过稳健的影响评估来评价保护战略的有效性, 并优化使用有限的资源。尽管评估保护影响的方法和工具不断出现, 但目前还没有标准化的方法来评估和比较物种恢复项目的影响和全球贡献。为弥补这一不足, 我们根据世界自然保护联盟(IUCN)衡量物种恢复的全球标准——物种绿色状况(Green Status of Species, GSS), 设计了一个评估框架。本研究旨在为保护项目实施的合作伙伴提供一种方法, 以评估其项目在促进全球物种恢复方面的有效性。我们改进了GSS评估中用于估算全球保护行动对某一物种影响的两种情景(当前反事实情景和未来无保护情景), 提出了一种新的评估方法, 称为“项目GSS”, 这是一种允许保护工作者估计保护项目相对于全球影响的过去影响和未来潜在影响的方法。为了确定在项目层面应用GSS方法的优势和局限性, 并为我们的方法改进收集概念证明, 我们利用16个物种恢复项目测试了该方法。在我们评估的项目中, 有7个可以利用“项目GSS”方法来确定项目行动在过去和未来对物种状况的影响。项目影响的可检测性以及与全球影响相比的项目相对影响, 均取决于项目建立的时间和实施范围(即包含的物种种群或分布区的占比)。我们的项目GSS评估框架可以为保护工作者提供一种标准化、直接、经济高效的方式, 来宣传保护成功和预期的未来影响。该框架得到的结果可与物种的全球恢复(保护遗产和保护影响)进行比较, 从而说明项目对整个物种恢复的贡献。**【翻译:胡怡思;审校:聂永刚】**

## INTRODUCTION

The current biodiversity crisis is worsening as anthropogenic pressures increase, so assessing the impact of actions taken to reverse biodiversity loss has never been more critical. Species recovery programs are essential in conservation, with over half of threatened species requiring species‐specific management interventions for their recovery (Bolam et al., 2023). It is crucial for practitioners to be able to objectively evaluate whether their work contributes to species recovery retrospectively and prospectively. Evaluating the effectiveness of conservation programs provides one the opportunity to replicate and improve programs that have had a positive impact on biodiversity and to adapt ineffective programs. To achieve this, the impact of species recovery programs needs to be evaluated and communicated across a range of stakeholders, including conservation nongovernmental organizations (NGOs), national and regional conservation agencies, government agencies, and funding bodies.

Many assessments of conservation impact focus on achieving institutional targets or goals, rather than objective biodiversity outcomes. It is also common for practitioners to base conservation success on input and output data, rather than impact data (Pressey et al., 2021). Although inputs (such as the financial, human, and technical resources invested in a program) are important for enabling conservation actions and outputs (such as the production of management plans or number of practitioners trained in certain techniques) demonstrate key intermediate steps, they do not necessarily lead to impact or long‐term change that can be attributed to conservation actions (Pressey et al., 2021; Salafsky et al., 2002). Furthermore, impact evaluation tools are not universal, and methods designed for evaluating the effectiveness of protected areas or community‐based conservation will not necessarily be applicable in evaluating species recovery (Brichieri‐Colombi et al., 2018; Leverington et al., 2008). Where efforts have been made to evaluate the impact of conservation on species recovery (Bolam et al., 2021; Butchart et al., 2006; Hoffmann et al., 2015, 2010; Young et al., 2014), there is too much variation among methods for results to be universally applicable, and they are not designed to assess the impact of specific programs. We sought to outline a standardized tool for evaluating the impact of single‐species recovery conservation programs that program partners can incorporate in their monitoring and evaluation plans.

We defined a species recovery program as a well‐defined, integrated set of conservation actions that have been planned and implemented over the long term and delivered in a specified geographic area by one organization or multiple organizations working in partnership to drive the recovery of a species. In cases where more than one organization is involved in the program, they are working toward a common vision and goals. Different organizations often form partnerships (e.g., government agencies collaborating with civil society organizations), and disentangling the impact of one organization from those of their partners is not practical (Young et al., 2014) and arguably not something to be encouraged. Therefore, we designed a program evaluation framework, not to evaluate the impact of an organization but rather the impact of a program. By following this framework, partners are able to report on the impacts of programs they are leading or involved in.

A large amount of literature exists to support program planning, prioritization, strategic decision‐making, and the use of adaptive management in conservation (Bolam et al., 2019; CMP, 2020; Gregory et al., 2012; Runge, 2011; Runge et al., 2011). The need to incorporate monitoring and evaluation of results to address uncertainty around the performance of conservation interventions and adapt management plans as needed is widely recognized (Baylis et al., 2016; Runge, 2011; Salafsky & Margoluis, 2003). Despite the importance of monitoring and evaluation of impact, it is still common for this step to be delivered poorly or in a nonuniform way (Buxton et al., 2020; Curzon & Kontoleon, 2016; Dixon et al., 2019; Wahlén, 2014), resulting in missed opportunities to learn from experience and improve program effectiveness to improve biodiversity outcomes (Wahlén, 2014).

A plethora of methods is available to assess the impact of conservation programs, but there are important constraints to be considered. Monitoring of species numbers and trends can be useful indicators of conservation success (Mazaris et al., 2017); however, they may not always effectively separate the effect of conservation actions from other confounding factors (Ferraro, 2009). To address this, experimental methods, such as randomized controlled trials (Grillos et al., 2019), before–after control–intervention (Conner et al., 2016), and quasi‐experimental methods, such as matching (Miteva & Pattanayak, 2021), have been used to robustly evaluate program impact. These approaches provide evidence from control or matched groups demonstrating outcomes in the absence of conservation interventions, allowing the attribution of actions to effects and providing the most defensible impact evaluation results (Baylis et al., 2016). However, experimental impact evaluation is rarely used in conservation (Ferraro et al., 2023; Schleicher et al., 2020). A lack of suitable control sites, legislative restrictions, and ethical implications of withholding support from control groups can hinder experimental evaluation (Baylis et al., 2016; Jellesmark et al., 2021; Pynegar et al., 2021). It can be costly to collect experimental and quasi‐experimental evidence (Grace, Akçakaya, Bull, et al., 2021), and the complexity of natural systems, alongside the scale of delivery and desired outcomes of conservation actions, can complicate experimental design (Baylis et al., 2016).

Due to these barriers, it has become increasingly common in conservation to use alternative, inferential approaches to quantify the impact of past conservation actions (Bolam et al., 2021; Bull et al., 2021; Butchart et al., 2006; Hoffmann et al., 2015, 2010; McMurdo Hamilton et al., 2023; Young et al., 2014). Using such methods involves generation of a hypothetical counterfactual scenario—an alternative scenario in which conservation efforts did not take place—that is compared with the observed outcomes (Grace, Akçakaya, Bull, et al., 2021). A range of evidence, including species status reports, trend data, expert elicitation, and population modeling, can be used to generate inference‐based counterfactual scenarios (Grace, Akçakaya, Bull, et al., 2021). Such approaches negate the need to gather experimental evidence and are therefore more cost‐effective and, providing a suitable evidence base is available, able to provide results more quickly. Several studies have used counterfactual methods to assess conservation impact as measured by the International Union for Conservation of Nature (IUCN) for their Red List of Threatened Species (hereafter red list) and Red List Index. By comparing a counterfactual red list category with the observed red list category, the impact on species recovery for collective conservation efforts across specified taxa or by a specific organization or partnership can be quantified (Bolam et al., 2021; Butchart et al., 2006; Hoffmann et al., 2015, 2010; Young et al., 2014). However, these and other counterfactual studies are not designed specifically to evaluate the impact of species recovery programs, and no method is available to achieve this program evaluation consistently.

We sought to address this lack of a comparable method by adapting the methods of the recently developed IUCN Green Status of Species (GSS), IUCN's global standard for measuring species recovery (IUCN, 2021a), to evaluate the contribution of specific conservation programs to the global recovery of a species. The GSS uses inferential counterfactual analyses to evaluate the impact of past conservation on a species’ progress toward recovery. In addition to evaluating past conservation impact, the GSS includes a metric of projected future impact to gauge planned actions’ likely contribution to species recovery and to inform conservation planning (Akçakaya et al., 2018).

**FIGURE 1:**
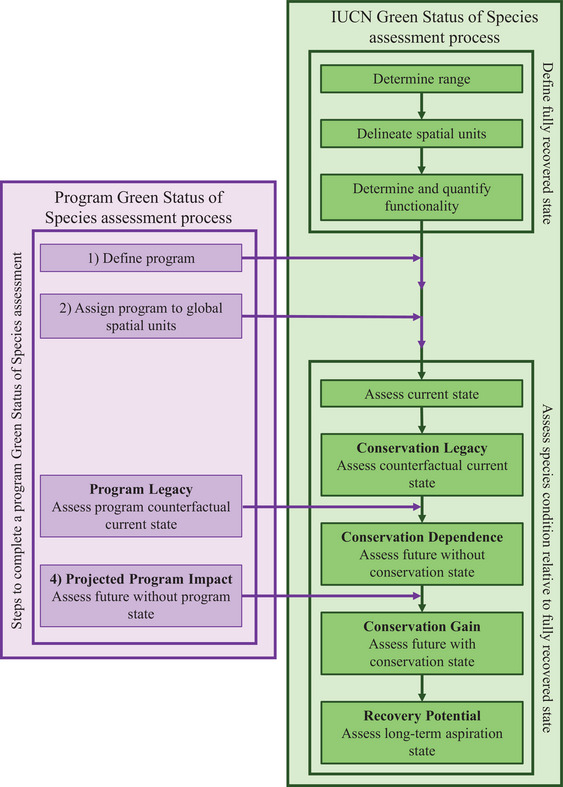
How the process of conducting a Green Status of Species (GSS) assessment of a program differs from a global International Union for Conservation of Nature (IUCN) GSS assessment (green, steps required for a global IUCN GSS assessment, completed prior to attempting a program assessment; purple, steps required for a program GSS assessment). For a detailed description of how each step in the IUCN GSS assessment is completed, refer to the *Background and Guidelines for the IUCN Green Status of Species* (IUCN GSSWG, 2024).

Launched in 2021, the GSS was developed as a complementary part of the red list (Akçakaya et al., 2018; Grace, Akçakaya, Bennett, et al., 2021). By considering conservation impact in terms of progress toward species recovery, the GSS aims to go beyond the traditional goals of maintaining species’ viability and preventing extinction by incentivizing conservation to reach levels that maintain a species’ function within the ecosystem (Akçakaya et al., 2020; Grace, Akçakaya, Bennett, et al., 2021). The GSS defines a fully recovered species as “viable and ecologically functional throughout its indigenous and expected additional range,” which is assessed by assigning states (absent, present, viable, or functional) to each part of a species’ range (called spatial units) (Grace, Akçakaya, Bennett, et al., 2021; Grace, Timmins, et al., 2021; IUCN, 2021a; Figure 1; Appendix S1). As well as reporting a species’ current status relative to being fully recovered (its species recovery score), the GSS can produce conservation impact metrics documenting past and expected future benefits of conservation actions on recovery (IUCN, 2021a) (Figure 1; Appendix S1).

Since its launch, the GSS has been promoted as a global standard for assessing species recovery in the red list strategic plan (IUCN, 2021b). The number of published assessments continues to increase, and there is a target for completion of 5000 by 2030 (IUCN, 2021b, 2025). A wide range of species across diverse taxa, including birds, reptiles, amphibians, trees, fishes, invertebrates, and mammals, has already been assessed under this framework to evaluate species recovery status and the impacts of conservation (Cano‐Alonso et al., 2023; Cogoni et al., 2022; Grace, Akçakaya, Bennett, et al., 2021). In line with the defined scope of the GSS Standard (IUCN, 2021a), all assessments have been at the global level and considered the combined effects of all conservation action on a species’ recovery.

One of the aims of the GSS is to incentivize ambitious conservation action for species recovery. Although the global GSS approach recognizes the combined impact of past achievements, highlights species dependent on continued actions, and forecasts expected gains from further combined action, conservation practitioners often seek to understand the contribution of their specific programmatic interventions to these goals and aim to prioritize conservation actions (Lloyd et al., 2023). We devised methods for adapting the GSS framework to produce an objective and standardized method that can be used by conservation practitioners to assess the impact of a specific conservation program on the global recovery of a species.

## METHODS

To identify the strengths and limitations of applying the GSS method at the program level and to gather proof of concept for this adaptation, we tested our proposed method on 16 species recovery programs (Table 1). Case studies were selected to represent a range of taxonomic groups, species generation lengths, intervention types, geographical scopes, and program durations.

**TABLE 1:** Summary of the 16 species recovery programs assessed using the proposed program Green Status of Species (GSS) method, including the program scope as indicated by the number of spatial units affected by the program compared with the number of spatial units identified in the International Union for Conservation of Nature (IUCN) GSS assessment.^a^

Species	Program	Number of spatial units^b^		Program details^c^	
		Global assessment	Program assessment	Conservation action	Number of years since program started
Telfair's skink (*Leiolopisma telfairii*)	Round Island restoration	14	3	Invasive species control; biosecurity; habitat restoration; protected area management; captive breeding; reintroduction	48
California condor (*Gymnogyps californianus*)	Baja California reintroduction site management	5	1	Reintroduction; postrelease monitoring; population monitoring; awareness and communication (engagement with hunters); lead poisoning mitigation	17
California condor (*Gymnogyps californianus*)	Breeding program	5	5	Captive breeding; studbook management; reintroduction; genetic research	44
Bolson tortoise (*Gopherus flavomarginatus*)	Breeding program	4	3	Reintroduction; benign introduction (to expected additional range); captive breeding (headstarting); population monitoring	17
Bolson tortoise (*Gopherus flavomarginatus*)	Biosphere reserve	4	1	Land acquisition tor protection; site management; erosion control and grassland management; awareness raising and communication (encourage sustainable practices)	7
Large antlered muntjac (*Muntiacus vuquangensis*)	Protection of species in the Annamite Mountains	3	3	Protected area expansion; site or area management; awareness and communication (understand social attitudes toward snaring); snare removal; training (community snare removal teams); monitoring; captive breeding (future); reintroductions (future)	10
Torrey pine (*Pinus torreyana*)	Torrey Pine State Park Reserve genetic study	1	1	Protected area management; invasive species control; wildfire mitigation; reintroduction; captive breeding; training; awareness raising; subnational legislation	6
Annamite striped rabbit (*Nesolagus timminsi*)	Protection of species in the Annamite Mountains	3	3	Protected area expansion; site or area management; awareness and communication (understand social attitudes toward snaring); snare removal; training (community snare removal teams); monitoring; captive breeding (future); reintroductions (future)	10
Scimitar horned oryx (*Oryx dammah*)	Scimitar‐horned oryx reintroduction program	8	1	Reintroduction	10
Hainan gibbon (*Nomascus hainanus*)	Supporting effective management through research and capacity building	1	1	Awareness raising and communication (promotion as flagship species); formal education (training on research methods); training (formal action planning)	13
Mountain yellow‐legged frog (*Rana muscosa*)	Northern populations	5	1	Reintroduction; captive breeding; invasive species control; disease mitigation	28
Mountain yellow‐legged frog (*Rana muscosa*)	Southern populations	5	4	Reintroduction; captive breeding; invasive species control; disease mitigation	17
Madagascar big‐headed turtle (*Erymnochelys madagascariensis*)	Rere conservation program	12	6	Support creation of protected areas; nest protections; support site management by local communities; invasive species control; habitat restoration; captive breeding; headstarting; translocation; training (nest protection, monitoring, alternative food resources); awareness raising (flagship wetland species, how to treat bycatch, festival)	25
Botsford's leaf litter frog (*Leptobrachella botsfordi*)	Vietnam frog conservation program	3	3	Research into species ecology and threats that is applicable across the entire range. Targeted actions only in one spatial unite: awareness raising (share species to wider audience); training (amphibian research and conservation methods, adopting amphibian friendly behaviors); site/area management (working with stakeholders to minimize habitat destruction)	8
Sterling's toothed toad (*Oreolalax sterlingae*)	Vietnam frog conservation program	3	3	Research into species ecology and threats, that is applicable across the entire range. Targeted actions only in one spatial unit: awareness raising (share species to wider audience); training (amphibian research and conservation methods, adopting amphibian friendly behaviors); site/area management (working with stakeholders to minimize habitat destruction)	8
Burmese star tortoise (*Geochelone platynota*)	Burmese star tortoise reintroduction program	4	2	Captive breeding; reintroduction; public engagement around release sites	24

^a^ Global IUCN GSS results are preliminary because they have not undergone the full IUCN review process, but all global assessments were facilitated by a member of the IUCN Green Status of Species (GSS)–Species Survival Commission Integration Task Force to ensure consistent application of the GSS methodology.

^b^ Spatial units used in the in the global and program GSS assessments.

^c^ Actions conducted within the program and the number of years since the program began.

The purpose of our proposed GSS assessment for conservation programs was to delineate the impact of a program on the global recovery of a species. For this reason, a global GSS assessment must be completed using the GSS Standard (IUCN, 2021a) prior to evaluating a program's impact. The global assessment considers the impact of all conservation actions and nonconservation factors (e.g. climate, geopolitical conflict) on a species’ recovery. Once a preliminary global assessment is complete, the focal program's contribution to the global recovery of a species can be evaluated by considering scenarios without the focal program while accounting for effects of nonfocal programs, broader conservation (e.g., legislation or protected areas establishment, though in some cases these may be program actions), and nonconservation factors (Figure 1).

We conducted preliminary global assessments for each case study species before undertaking any program GSS assessments (Table 1). The process of completing a global GSS assessment is summarized in Figure 1 and Appendix S1. In short, the species’ range prior to major human impacts (indigenous range) was determined. For example, the California condor's (*Gymnogyps californianus*) indigenous range was western North America (California, Oregon, Nevada, Washington, Idaho, Arizona, and Baja California). The indigenous range was then divided into spatial units, representing areas of similar conservation importance. Identification of spatial units allowed the assessment to detect variation in recovery and incentivize conservation across the species’ range. For example, the California condor's indigenous range was divided into 5 spatial units (Table 1) based on subpopulations (Hermes & Vilchis, 2024). The condition of the species in each spatial unit at the time of assessment was then assessed and assigned to one of 4 states (absent, present, viable, or functional) (details below and in Appendix S1). These states were used to calculate the current status of the species (species recovery score). The condition in each spatial unit was also assessed under 4 hypothetical scenarios (counterfactual current, short‐term [10‐year] future with conservation, short‐term future without conservation, and long‐term [100‐year] future with conservation). The states estimated under these scenarios were used to calculate 4 conservation impact metrics pertaining to the past (conservation legacy) and expected future (conservation gain, conservation dependence, recovery potential) impacts of conservation (Figure 1; Appendix S1).

To reduce bias and subjectivity, guidance for creating robust counterfactuals and providing inferential evidence to estimate conservation impact was applied (Grace, Akçakaya, Bull, et al., 2021; IUCN GSSWG, 2024). Information used to estimate states under hypothetical scenarios included consulting species experts on the expected outcomes under different scenarios; using experimental evidence where available; considering species trend data, population models, and evidence from proxy species; and using logical arguments to interrogate the assumptions underlying the observed or expected impacts of conservation actions. The guidance also advised on how to include external variables that may contribute to changes in species recovery scores (e.g., civil unrest) in the counterfactual. Following completion of the global GSS assessments, program GSS assessments were carried out to determine the contribution of 16 programs to the global recovery of their focal species.

We expected program impact to vary depending on various factors, such as the geographical scope of the program, establishment date, and types of conservation interventions (Table 1). We identified the partners contributing to and delivering the program (including, but not limited to, conservation NGOs, government bodies, academic institutions) and the timing of conservation actions delivered within the program (past, ongoing, or planned) to explore how delivery and results of actions may interact with each other. To ensure comparability and rigor, spatial units used at the program level were consistent with those assigned in the global GSS assessments.

**FIGURE 2:**
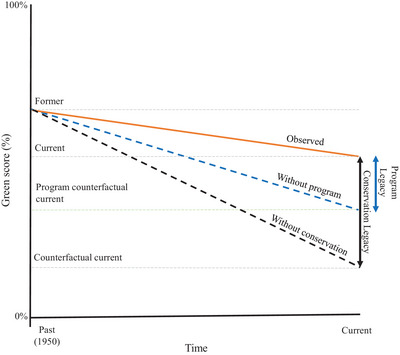
Past conservation impact at the global (black vertical arrow) and program (blue vertical arrow) level expressed as the change in International Union for Conservation of Nature (IUCN) green score for a species over time under different scenarios (orange, actual change in green score with all past conservation action; black dashed, counterfactual, or expected change in green score without past conservation action as calculated using the IUCN Green Status of Species [GSS]; blue dashed, program counterfactual, or expected change in green score without past conservation actions of the program being assessed but with all other conservation actions as calculated using the program GSS). The IUCN green score represents how far a species is from its fully recovered state, from 0%, extinct in the wild, to 100%, fully recovered (or not depleted) (IUCN, 2021).

In the global GSS assessments, the impact of past conservation actions on the recovery of a species was assessed by estimating what the current state of the species would be in each spatial unit if no conservation action had occurred to date (the counterfactual scenario). A similar approach was applied to estimate the program counterfactual. We estimated the state of the species within a spatial unit in the absence of the focal program's past conservation actions but including all other conservation actions (Figure 2). The resulting program legacy metric estimated the extent to which the focal program has contributed to the global conservation legacy. The program legacy will usually be smaller than the conservation legacy where there is more than one program contributing to the conservation of the species. However, in cases where the program is responsible for all legacy actions delivered, the values may be the same.

Calculating future program impact was more complex. The global GSS framework provided 3 metrics for quantifying the expected future impacts of conservation on species recovery. The conservation gain and conservation dependence metrics reflected the expected benefits of ongoing and planned conservation actions over the next 10 years, and the expected negative impacts of discontinuing them over the same time frame, respectively. Recovery potential quantified the maximum possible impact of conservation over the next 100 years.

In the global GSS assessment, conservation gain and conservation dependence were calculated by comparing scenarios estimating the state of the species in each spatial unit in 10 years, with and without the effects of conservation, to a baseline (by default, the current green score) (IUCN, 2021a). The 2 metrics can be summed to produce one single metric of conservation impact (Grace, Akçakaya, Bennett, et al., 2021; IUCN GSSWG, 2024). This combined approach was followed to evaluate the expected effects of a program over 10 years and produced a single metric of projected program impact. We used this approach because separating gain and dependence around a baseline was challenging when considering a program assessment because the effects of nonprogram conservation actions also needed to be considered. The use of the default current baseline led to inaccurate results of zero or negative program gain or dependence when changes resulting from interventions outside the program being assessed were not correctly accounted for.

As with the program legacy, the method for calculating future conservation impact was adapted to estimate species’ states with and without the effects of the focal program and resulted in the projected program impact. This included the effects of ongoing program activities already in place and activities planned for implementation in the short term. Thus, projected program impact was the difference between a future scenario with the program and one without the program. This straightforward calculation became complicated by the requirement to account for both ongoing and planned nonprogram conservation activities.

**FIGURE 3:**
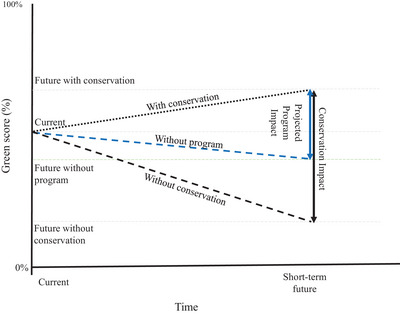
Future conservation impacts at the global (black vertical arrow) and program (blue vertical arrow) level expressed as the change in International Union for Conservation of Nature (IUCN) green score for a species over the short‐term future (10 years) under different scenarios (black dotted line, expected short‐term change if all conservation action continued, as calculated using the IUCN Green Status of Species [GSS]; black dashed line, expected short‐term change if all conservation action stopped, as calculated using the IUCN GSS; blue dashed line, expected short‐term change if conservation action of the program being assessed stopped but all other conservation continued, as calculated using the program GSS. The IUCN green score represents how close a species is to its fully recovered state, from 0%, extinct in the wild, to 100%, fully recovered (or not depleted) [IUCN, 2021]).

Two approaches accounted for nonprogram activities: include the activities in both scenarios (with and without the program) (Approach 1) or exclude them from both scenarios (Approach 2). We tested these approaches by applying them to 6 program case studies. A full description of testing and results is in Appendix S2. Based on this testing, it was determined that including nonprogram actions in both future scenarios was the most broadly applicable approach. Therefore, to calculate the projected program impact, the expected short‐term future state of the species within a spatial unit was estimated in the absence of the focal program's ongoing and planned conservation actions but including all other conservation actions (Figure 3). This was compared with the future with conservation scenario, as estimated in the global assessment, which includes all ongoing and planned conservation actions globally. The resulting projected program impact metric estimated the extent to which the focal program contributed to the global conservation impact.

Assessors might not always be aware of planned actions outside their programs. To address this, we applied a third approach (Approach 3) in which the effects of planned nonprogram conservation actions were not considered but active nonprogram actions were. This third method was less commonly used by our case study assessors and is expected to be used infrequently in general (discussed in Appendix S2).

At the global scale, the GSS also considers the long‐term impacts of conservation by assessing all possible conservation actions over 100 years unconstrained by finances but considering factors such as the species’ reproductive rate, climate change, and expected socioeconomic developments (Akçakaya et al., 2018). Although the long‐term contribution of programs was considered (Appendix S3), we were not aware of any programs for which actions were planned over a 100‐year time frame and plans were made without considering financial constraints. Therefore, we did not assess the programs at 100 years, and for most program‐level GSS assessments, it is not recommended to estimate the impacts of the focal program over a 100‐year time frame.

## RESULTS

**FIGURE 4:**
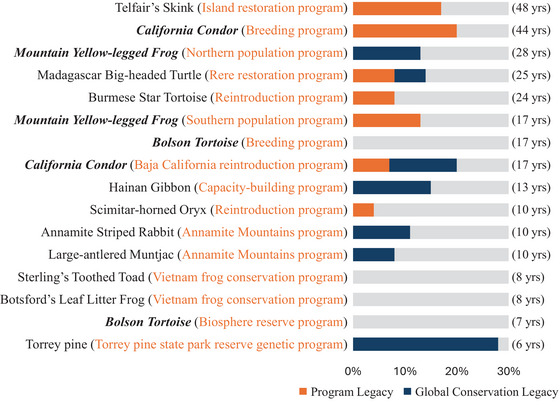
Comparison of the program legacy (orange, impact of past program actions for the species) with the global conservation legacy (blue, impact of all past conservation actions for the species) ordered from longest‐running to most recently started program (yrs, years; legacy percentage, difference between green scores with and without past conservation; only orange bar, program legacy is equal to the global conservation legacy; only blue bar, global conservation legacy but no program legacy; gray bar, no detectable global conservation or program legacy; bold italics, multiple programs evaluated). Results of global assessments have not been fully reviewed by International Union for Conservation of Nature (IUCN), but all assessments were facilitated by a member of the IUCN Green Status of Species–Species Survival Commission Integration Task Force to ensure consistent application of the Green Status methods.

**FIGURE 5:**
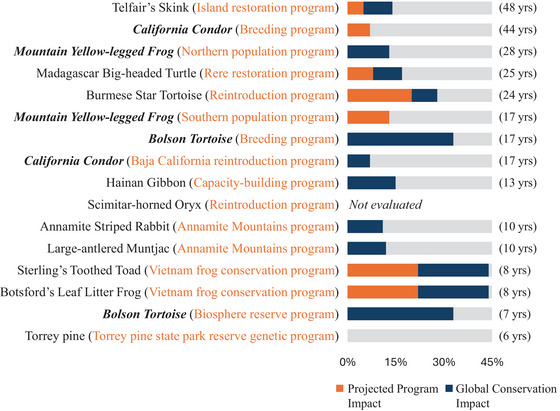
Comparison of the projected program impact (orange, estimated impact of ongoing program actions over the next 10 years) with the global conservation impact (blue, estimated impact of all ongoing conservation actions for the species over the next 10 years ordered from longest‐running to most recently started program; yrs, years; impact percentages, difference between green scores with and without future conservation actions; only orange bar, projected program impact equal to the global conservation impact; only blue bar, global conservation impact but no projected program impact; gray bar, no detectable global conservation or projected program impact over the next 10 years; bold italics, multiple programs evaluated). Results of global assessments have not been fully reviewed by International Union for Conservation of Nature (IUCN), but all assessments were facilitated by a member of the IUCN Green Status of Species–Species Survival Commission Integration Task Force to ensure consistent application of the Green Status methods. Assessors do not have to complete all scenarios in an assessment, so there is no conservation impact or projected program impact for the scimitar‐horned oryx assessment because only the past impact of the program was assessed.

Of the 16 assessed programs, we detected past impact in 7 programs and expected future impact in 7 programs (Figures 4 & 5). In some instances, the program legacy equaled the global conservation legacy, as seen in the case of the Telfair's skink (*Leiolopisma telfairii*), where one long‐running program was the sole program acting on the entire remaining range of the species. Also, a single reintroduction program facilitated the reestablishment of the extinct‐in‐the‐wild scimitar‐horned oryx (*Oryx dammah*) in part of its range. More commonly, the program legacy will be less than the conservation legacy—for example, the California condor reintroduction program in Baja California. This program represents on‐the‐ground work supporting reintroductions in 1 of 4 sites. Thus, the program legacy was based only on the Baja California site, but all 4 reintroduction sites contributed to the conservation legacy of the species.

There were 3 species—Bolson tortoise (*Gopherus flavomarginatus*), California condor, and southern mountain yellow‐legged frog (*Rana mucosa*)—for which we were able to assess multiple programs contributing to their recovery. In 2 of 3 instances, results were not additive toward the global scores. When assessing the 2 programs contributing to the global recovery of the Bolson tortoise, the sum of the projected program impact of these programs was less than the global conservation impact. For the California condor, the actions of the 2 programs assessed overlapped; thus, the sum of the program legacy from the 2 exceeded the conservation legacy. Potential reasons for the nonadditive nature of the program assessments are explored in the “DISCUSSION” section.

Our case studies showed 3 instances of a program acting in part of one or several spatial units, rather than the entire spatial unit. The Annamite striped rabbit (*Nesolagus timminsi*), large‐antlered muntjac (*Muntiacus vuquangensis*), and Madagascar big‐headed turtle (*Erymnochelys madagascariensis*) programs all had a geographic extent limited to a small number of protected areas in a larger spatial unit. Because states are assessed at the level of an entire spatial unit, program assessors must also consider the state of the species outside the program's sites when the geographical scope of the program only partially covers a spatial unit. In instances where multiple programs operate within a single spatial unit, the work of other programs will be included as other ongoing and planned actions. Such cases may benefit from using Approach 3 for calculating projected program impact (inclusion of only ongoing nonprogram actions [Appendix S2]) because assessors may not be equipped to estimate the impact of planned actions external to the focal program.

Our case studies covered programs that applied a range of interventions, including release of captive‐bred individuals, biosecurity, habitat restoration, research, education, and awareness raising (Table 1), to assess whether the impact of different intervention types was captured using the assessment framework. The framework proved capable of detecting impact across varied intervention types. For example, species recovery programs involving reintroductions commonly resulted in relatively high results for both program legacy and projected program impact, as demonstrated by the Telfair's skink, scimitar‐horned oryx, and California condor. In all 3 species, reintroductions resulted in changing the state of at least one spatial unit from absent to at least present. Without this conservation action, these species would have remained absent in these spatial units.

Many programs tested had some elements with less hands‐on species management actions and more of a focus on establishing an evidence base for effective species conservation, capacity building, and community work. The past actions of 2 species recovery programs, Botsford's leaf litter frog (*Leptobrachella botsfordi*) and Sterling's toothed toad (*Oreolalax sterlingae*), focused on research to inform evidence‐based recommendations for strict protection of critical breeding habitats and training, capacity building, and awareness raising with local stakeholders to minimize the impacts of tourism. Both programs expect improvement in the status of the species over the next decade.

In some cases, combinations of interventions were used in a single program (Table 1). Specific interventions are not assessed individually. Therefore, our assessment is more appropriate for determining whether a program as a whole has had or will have the desired effect on species recovery. If it does or will have the desired effect, then further investigation into which program actions were most critical to success can be undertaken.

An important factor potentially affecting whether a program delivered detectable past or future impact was how long the program had been running. Of our 16 cases, 9 had no detectable program legacy (Figure 4). Of these, the average amount of time since the establishment of the program was 11.9 years (SE 2.4) (Table 1; Figure 4). In contrast, the average time since the start of the program for the 7 examples with a program legacy was 26.4 years (5.0) (Table 1; Figure 4). Similarly, when assessing projected program impact, programs with a detectable effect on species recovery in the next decade had been established for an average of 24.9 years (5.6), compared with 13.5 years (2.4) for those with a projected program impact of zero (Table 1; Figure 5).

## DISCUSSION

Our aim was to explore the potential benefits and limitations of adapting the GSS framework to assess the impact of individual conservation programs on species recovery and to provide conservation practitioners with a standardized way to measure the past and potential future impact of their work. The GSS was originally developed to address a need for a standardized measure of species recovery across the entire range of a species (Akçakaya et al., 2018). The adaptation we devised and trialed is an important next step in using the GSS as a global standard for measuring species recovery and addressing the gap in standardized measures at the program level (Young et al., 2014). Due to the collaborative nature of conservation, it is generally impossible to disaggregate the relative contributions of different partners, and attempting to do so risks causing conflict (Young et al., 2014). Thus, the purpose of the approach presented here is to assess the impact of a species recovery program as a whole, acknowledging all contributing partner organizations.

Although conservation actions are having a positive impact on global biodiversity (Langhammer et al., 2024), there is a need for more and better counterfactual studies to determine conservation impact. Despite the overall positive contribution of conservation to biodiversity, interventions need to be expanded to a scale sufficient to reverse the global biodiversity crisis (Langhammer et al., 2024). Using the GSS framework will allow practitioners to evaluate the past and expected future impacts of programs, which can be valuable for adaptive management of programs and communications with funders and stakeholders. Although the single‐species assessments provided by the program GSS are not suitable for the evaluation of all types of conservation programs, single‐species‐focused programs are common (Bolam et al., 2021; Burgess et al., 2019; Young et al., 2014) and, in many cases, are necessary due to the need for targeted, species‐specific actions for the recovery of threatened species (Bolam et al., 2023). Flagship species can attract funding, umbrella species can drive habitat restoration, and national environmental legislation, such as the US Endangered Species Act, focuses on single‐species management plans (Runge et al., 2019; Thomas‐Walters & Raihani, 2017). The program GSS is designed to be a high‐level indicator complementing more regular monitoring of program progress toward goals and objectives. Through periodic program GSS evaluations, practitioners can go beyond assessing whether activities are on track to meet objectives set within a program plan to assessing the impact of their species recovery programs in a global context and determining whether the impact of a program is sufficient to see progress toward species recovery at the global scale.

### Communication and use of results

In developing this framework, we strove for consistency with the GSS Global Standard (IUCN, 2021a) to ensure that program GSS assessments can be directly compared with global assessments. However, one key difference pertains to the conservation impact metric outputs: the program GSS combines conservation gain and conservation dependence in a single projected program impact metric. This means that when communicating results comparing a program assessment with a global assessment, projected program impact should be communicated in comparison with conservation impact, which is the combination of conservation dependence and conservation gain (Grace, Akçakaya, Bennett, et al., 2021; IUCN GSSWG, 2024).

As demonstrated by our case studies, our suggested approach has the potential to quantify the impact of programs in a way that can be compared with the global recovery of a species. Examples of high program legacy can be used to celebrate program impact achieved to date and be shared with relevant stakeholders and supporters. Program legacy can be used to gain support and future funding by enabling conservation programs to demonstrate their capabilities based on a measure of the impact achieved by their programs in the past. Additionally, the program GSS measures the projected program impact by quantifying what the program is expected to achieve in the future given support and resourcing for ongoing and planned conservation actions. Although the GSS is not designed to be a prioritization tool (Akçakaya et al., 2018), the information gleaned from this objective assessment may be used to inform management decisions, such as whether to commence, continue, or expand certain programs, because practitioners can consider the expected level of future impact alongside other important factors, such as costs, stakeholder interests, and impact on other species (Game et al., 2013). The use of the GSS as part of a cost–benefit evaluation has been explored previously by Lloyd et al. (2023). The first stage of their assessment, focused specifically on the impact of translocation programs, is to determine the status of the focal species with and without translocations. This same approach could be applied for a range of interventions based on the results of the program GSS as the first step in the cost–benefit analysis workflow outlined in Lloyd et al. (2023).

If evaluation of a particular program does not detect any impact at the global scale, results provide an opportunity for practitioners to critically evaluate the reason for the lack of measurable impact. In some instances, this may be an artifact of species biology (e.g., slow life history) or how recently programs have been established (see the “FUTURE DIRECTIONS” section), but it may also be related to the program scope. Our case studies demonstrated that results from program GSS assessments may help identify instances where programs are not working at sufficient scale to impact global species recovery. In the case of the Madagascar big‐headed turtle, the focal program was restricted to a small number of protected areas within a larger spatial unit (defined by watershed). There were spatial units where despite conservation successes of the program (stable and increasing populations in the focal lake or river), continued deterioration elsewhere in the spatial unit resulted in these successes having limited impact on the species’ overall recovery within the spatial unit. Such results could be valuable in helping determine where programs need to be scaled up, either by expanding the geographical scope or increasing capacity, possibly through partnerships, to deliver impact at the scale required for the species. This aligns with the findings of Langhammer et al. (2024) that a major challenge facing conservationists is scaling up conservation actions sufficiently to address the biodiversity crisis and should be considered when planning and evaluating programs.

The aim of the program assessment is to determine and communicate a program's contribution to the global recovery of a species by comparing program impact metrics with global conservation impact metrics. Due to nonprogram actions, or interventions that are not independent of each other, the results of all program impact metrics are not necessarily expected to add up to the corresponding global conservation impact metrics, as illustrated in the Bolson tortoise and California condor case studies. When assessing the 2 programs contributing to the global recovery of the Bolson tortoise, the sum of the projected program impact of these programs was less than the global conservation impact due to important nonprogram conservation actions: a key protected area preserving habitat for most of the population and legal protection preventing poaching. In the case of the California condor, the actions of the 2 programs assessed overlapped. The overarching breeding program provides birds for reintroductions, and on‐the‐ground programs mitigate threats and support reintroductions to 4 sites. When considering the California condor breeding program and the on‐the‐ground program supporting reintroductions in Baja California, the sum of the program legacy from the 2 exceeded the conservation legacy because the interventions were not independent. Birds born in the breeding program include the individuals introduced in the Baja California program. For this reason, program impact metrics cannot be communicated as contributing a specific percentage or proportion of the conservation impact metrics. Rather, the 2 results should be presented simultaneously.

### Limitations

Program assessments may be more susceptible to subjectivity and bias than global assessments, given the desire to demonstrate high program legacy or projected program impact to donors and stakeholders. The guidance on determining robust inferences outlined in the GSS guidelines and associated publications (Grace, Akçakaya, Bull, et al., 2021; IUCN GSSWG, 2024) should be applied at the program level, and we strongly discourage attempting a program GSS assessment until a reviewed global assessment for the species is available. This ensures that the elements of the global GSS assessment (which underpin the program GSS assessment) have been applied correctly, as per the GSS Standard (IUCN, 2021a). Using the justifications and evidence from a reviewed GSS assessment to guide assessors when evaluating scenarios in the program assessment can help reduce the risk of subjective justifications. Another factor that could introduce bias is the selection of spatial units, which is mitigated by the requirement that the same spatial units are used in a program GSS assessment as at the global level. As well as being necessary to allow for comparison between global and program GSS assessments, this consistency avoids assessors selecting inappropriate spatial units because of a lack of familiarity with methods or intentional manipulation of the system to increase perceived program impact. In instances where global assessments have not been reviewed, it should be made clear that program assessment is based on a preliminary global assessment. Although there is advice in place to minimize bias, unlike global GSS assessments, which undergo an independent peer‐review process prior to publication on the IUNC Red List website and must conform with strict supporting documentation requirements, program GSS assessments will be for internal use and not published on the red list website, so they will not undergo this review process. Therefore, the onus will fall heavily on program assessors to institute and ensure the necessary level of internal review, and it is highly recommended that multiple assessors from across the program partnership be involved in the assessment.

A current perceived limitation to the uptake of the program GSS for impact evaluation is the relatively low number of global assessments published (IUCN, 2025). The GSS is still in its infancy compared with the red list, but the number of assessments continues to increase, with the number published and the number in preparation and review per year having more than trebled since 2022.

Although we expect to see the number of published GSS assessments greatly increase in the coming years, we also highlight that completion of program GSS assessments for partners to evaluate the impact of their programs does not require a high number of assessments. The purpose of this tool is to allow partnerships to understand the value of long‐term and significant investment in species recovery programs and how they contribute to global species recovery. Thus, in many instances, the limitation is the availability of a single‐species global assessment that adheres to all requirements of the published GSS guidance (IUCN, 2021a; IUCN GSSWG, 2024). Often program assessors will be well placed to complete a preliminary draft of a global GSS assessment, and with the target of completing 5000 assessments by 2030, there is incentive for SSC specialist groups to provide review and oversight through collaboration with program assessors. There may be instances where the aim is to complete multiple program assessments, similar to previous work where an assessment of all long‐term programs contributed to by Durrell Wildlife Conservation Trust and their partners was conducted (Young et al., 2014). In such cases, more global assessments would be required. In the example from Young et al. (2014), 17 target species were considered, so an equivalent assessment with the program GSS would therefore only require 17 global assessments.

### Future directions

To further understand the range of appropriate uses for the program GSS, receive feedback on the assessment process, and provide additional support for application, we continue to work on its development. This entails more extensive testing on a range of programs with varying characteristics to better understand its utility and refine guidance for assessors. For this wider testing, which is already underway, the assessors are provided with standardized materials including an online assessment workbook, associated guidelines, and step‐by‐step instructions (ICCS, 2024; IUCN GSSWG, 2024). In addition to the resources provided, online training is available (Grace et al., 2022).

Several of our case studies highlight a potential limitation of our proposed method for assessing future program impact because a 10‐year horizon is often insufficient to detect the effects of conservation. Species recovery commonly takes decades, rather than years, because species rarely respond to conservation actions immediately (Watts et al., 2020). Among our case studies, the programs with a projected program impact >0 (average time since program start = 24.9 years) had been in place nearly twice as long as those with no projected program impact (average = 13.5 years). There are several potential explanations for the absence of a demonstrable effect on the species with a 10‐year time span, including the long generation length of the assessed species; interventions not delivering immediate conservation impact but focusing on building toward future conservation (e.g., capacity building, research); the time taken for reintroduced species to be considered wild (based on the red list definition [IUCN, 2022]); and programs and interventions being at a relatively early stage, requiring time to develop and affect detectable and measurable change. Previous work in which the IUCN Red List Index was used to evaluate organizational impact reported the average time between the start of conservation interventions and the year of the first downlisting of the target species was 16.3 years (range 11–25 years) (Young et al., 2014). When assessing the recovery programs of critically endangered bird species in Mauritius, it could take up to 30 years to restore populations to a point of requiring minimal management (Jones, 2004). Different conservation interventions are expected to yield different levels of impact over different time frames. Our aim was to produce a method that can be used to evaluate all types of programs effectively. Because it is becoming increasingly common for programs and governments to consider long‐term visions for species recovery (Russell et al., 2015; Sanderson et al., 2008), we suggest that it may be beneficial to assess an additional time frame between the 10‐ and 100‐year points to make this tool more applicable to more recently established programs and programs with interventions where results are not realized immediately. This temporal aspect of program impact is something we aim to understand further through the ongoing wider testing of the program GSS that will allow us to continue to develop appropriate guidance.

Although our program legacy results demonstrated the past impact of a program's collective actions, these results alone do not provide conservationists with evidence of how individual actions associated with the program did (or did not) contribute to that impact. Assessors may therefore choose to consider the program legacy alongside additional supporting information to understand the impact of specific actions delivered under the program. Justifications in the assessment can provide data to contextualize specific interventions. For example, for the Telfair's skink, data presented in the justification showed that biosecurity preventing invasion of islands by non‐native predators is important for the continued success of the species. Data on the approximate rate of offshore island invasions by non‐native fauna per year and evidence of the disastrous impact on reptile populations from previous invasions were used to inform the expected future status of the species without the conservation program (ICCS, 2024). Alternatively, use of logical arguments to evaluate assumptions associated with attributing change to specific actions, for example, through a theory of change, can help determine the most likely states in scenarios during a GSS assessment.

By considering the assumptions and evidence associated with how an action is expected to influence a threat, both indirectly and directly, assessors can present a better supported argument for how a program action has resulted in a change (Margoluis et al., 2013). Such information could be used alongside the program legacy result to further understand the relative importance of different actions delivered within a program. Alternatively, methods similar to those already implemented with the IUCN Red List Index to distinguish the primary threats driving a decline or actions driving improvement (Bubb et al., 2009; McGeoch et al., 2010) could be adopted to assess the most important program conservation actions. It is important to consider, however, that such an approach will not always be appropriate because some programs may only be effective because of the suite of actions delivered together (e.g., the intervention of reintroduction may not be possible without the intervention of invasive species control).

The program GSS approach may also be applied to evaluate programs acting on wide‐ranging species at the country level, for example, the Thai Government's tiger recovery program or the Nepalese government's gharial recovery program. In both instances, the geographic extent of the program (Thailand and Nepal, respectively) focuses on one of multiple range countries and incorporates parts of multiple spatial units that are within the country boundaries. Assessments at this scale could provide useful results for countries to assess conservation impact, but testing with a range of examples of country‐level programs is required to better understand this potential. Finally, periodic reevaluation of programs with this method could help compare how observed changes relate to those predicted in the program assessment and determine whether a projected program impact has been achieved.

When a larger number of programs have been tested, we will refine this method of evaluating program impact. We invite contributions to the continued testing effort and feedback on this framework to support us in developing robust guidance for applying the GSS at the program level.

## Supporting information



Supporting Information
