# Supplementary material for: Evaluating past and future contributions of conservation programs to species recovery
Source: Conserv Biol. 2025 Nov 25;40(2):e70183. doi: 10.1111/cobi.70183 (PMC13036293; doi:10.1111/cobi.70183)
Supplement: Supplementary file 1 — Supporting Information [file COBI-40-e70183-s001.docx]

## Appendix S1: Summary of the IUCN Green Status of Species methods


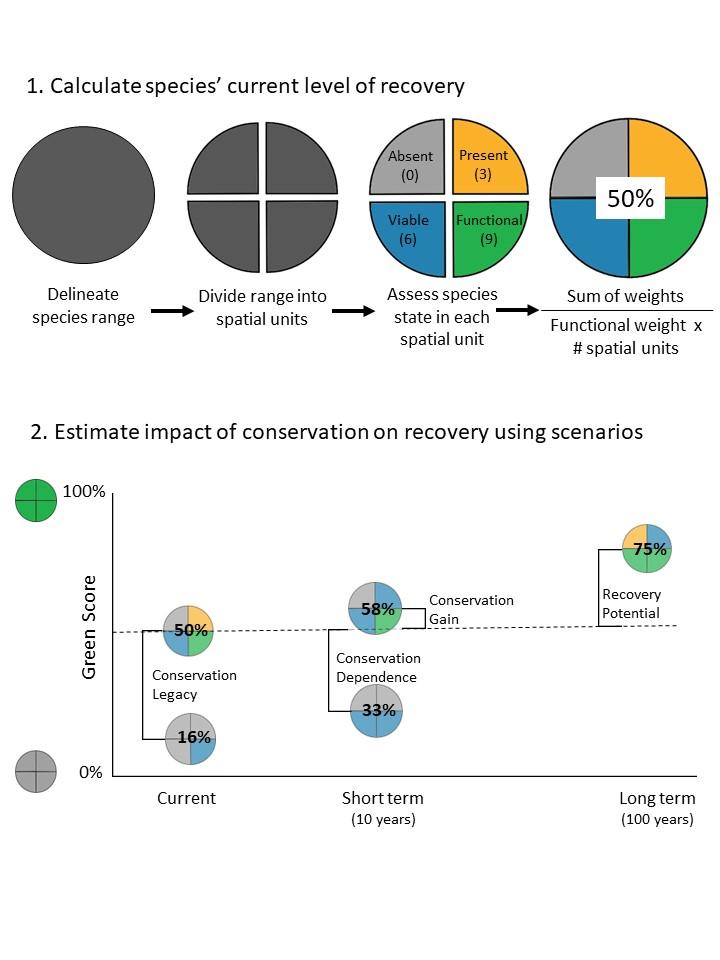


Figure S1: Summary of the assessment process and outputs for the IUCN Green Status of Species, based on Figure 1 in Grace et al. (2021c) (reproduced with permission from authors). A species’ indigenous range, i.e., range prior to major human impacts, and any expected additional range are determined, then divided into spatial units to capture variation in recovery and incentivize recovery across the range. The current status of the species is assessed in each spatial unit and one of four states is assigned (absent (grey), present (blue), viable (orange), functional (green)). Each state has a different weighting, as indicated in parentheses which is used to calculate the current status of the species (or species recovery score, SRS) relative to the fully recovered state (all spatial units are functional; SRS = 100%). Conservation impact metrics (conservation legacy, conservation gain, conservation dependence and recovery potential) are determined based on the difference between the current SRS and hypothetical scenarios with and without conservation, both past (conservation legacy) and future (conservation gain, conservation dependence, recovery potential). This figure shows data for a hypothetical species with four spatial units. This is a simplified example that does not take into account all possible cases; for a full account, see IUCN 2021.

## Appendix S2: Testing approaches for calculating projected program impact

Here, we review the two approaches to account for non-program activities in the 10-year projected scenarios. These approaches are including non-program action in both scenarios (with and without the program) or excluding them from both scenarios. We demonstrate that, despite the difference in the inclusion or exclusion of non-program conservation actions, if the effects of program and non-program actions can be considered independently of each other, they produce equivalent results.

### Methods

Approach 1 includes all non-program actions. This method estimates the scenario where all focal program activities cease immediately, while all other conservation continue as planned. This option includes all ongoing and planned non-program conservation actions in both short-term (10-year) future scenarios (with program and without program) (Figure S2a). Assessors using this method only need to estimate species’ states under one new scenario – future without program, where all non-program conservation actions continue but those of the focal program do not. Consequently, the future with program scenario is the same as the global future with conservation scenario (Table S1).

Approach 2 excludes all non-program actions. This method compares a future with no conservation whatsoever to a future where the only conservation is attributed to the focal program. This option excludes the effects of all other (non-program) conservation actions, both ongoing and planned, from both short-term future scenarios (Figure S2b). Assessors using this method only need to estimate species’ states under one new scenario— future with program only, which excludes all actions except those of the focal program. Consequently, the future without program scenario is the same as the global future without conservation scenario (Table S1).

### Results

We hypothesized that the two approaches to calculating projected program should produce equivalent results (Table S1). To test this hypothesis, we calculated projected program impact for six of our 16 test programs using both methods (Table S2).

The two approaches produced different estimates of projected program impact in two of the six cases tested (Table S2). Upon reviewing the reasons for this discrepancy, it became clear that a key assumption of our hypothesis was violated. This was the assumption that conservation interventions in the focal program are independent of those occurring outside the focal program, allowing one to be considered without the other. However, this is assumption is not valid, as most programs likely rely on enabling conservation actions external to the program. Therefore, it was often challenging to exclude non-program actions (approach 2).

Reintroduction programs provide a useful example for this interdependence between program and non-program actions. For a reintroduction to be successful, it is essential to mitigate the threats which initially resulted in species extirpation (IUCN/SSC 2013). In the case of the scimitar-horned oryx (*Oryx dammah*), which was declared extinct in the wild in 2000 (IUCN SSC Antelope Specialist Group 2016), this species is once again present in part of its native range following a reintroduction program. The success of the reintroduction program relied on having a suitable site to reintroduce the species (Ouadi Rimé-Ouadi Achim Faunal Reserve in Chad), without which no reintroduction would have occurred. Therefore, using approach 2 and assessing the state of the species in a scenario with the program only and no other conservation is not practical. This is because the program would not have been implemented independently of other background conservation actions (in this case, the associated management the protected area). However, as the protected area was not established as part of the program, or for the sole purpose of protecting the scimitar-horned oryx, this other conservation action would have continued regardless of the presence or absence of the focal program (inclusion of all non-program actions). This makes approach 1 a more tractable option for calculating projected program impact.

In our case studies, we encountered numerous instances where program actions would be ineffective, or substantially less effective, in the absence of external conservation actions implemented outside the program. For example, reintroductions of species threatened by poaching would be ineffective without their legal protection; translocations and reintroductions of species threatened by habitat loss and degradation require protected areas for suitable release sites; actions to expand protected areas require an established protected area; and island biosecurity actions protecting native fauna from invasive predators would be much more difficult to implement if sites were not protected areas with restricted access. In all cases, investing valuable conservation resources into programs without the enabling actions external to the program first being in place would be ineffective, and we would not observe genuine examples of programs delivered independently of all other conservation. Consequently, it was determined that, despite being a plausible concept, approach 2 was not a broadly applicable method for assessing program impact using the GSS in real-world cases, therefore we do not advocate the use of approach 2.

### Separating ongoing and planned conservation actions

A third approach to calculating projected program impact was tested in addition to those outlined above. This approach is slightly more complicated as it requires separating ongoing and planned conservation actions outside the program, but has the benefit that assessors do not need to predict the outcome of planned actions outside the focal program of which they have less expertise.

Approach 3 includes only ongoing non-program actions. For both short-term (10 year) future scenarios (with program and without program), include ongoing non-program conservation actions and exclude planned non-program conservation actions (Figure S1c)

This option includes the effects of ongoing non-program conservation actions, and excludes the effects of planned non-program conservation actions. It may appear more complicated, but it may be the most practical in some cases. For example, it may be simpler to exclude the effects of planned non-program conservation actions, rather than trying to predict the impact of a conservation action that is not yet operational. In particular, assessors may not feel confident predicting the outcomes of planned interventions falling outside of their focal program of work. In summary, this method estimates what would happen if all focal ongoing program activities were stopped today, and all other non-program conservation continued as is, but no additional planned actions outside the program started.

When testing the different approaches of calculating projected program impact, including non-program conservation actions produced consistent results, whether planned actions were considered or not, therefore approach 1 and approach 3 were consistent in every program tested (Table S2). When testing the program GSS methods more widely, there was not commonly a need for the separation of planned and ongoing non-program actions, therefore this method was used the least.

Table S1. Comparison of the 3 approaches to calculate projected program impact.*^a^*

|  |  | Program actions | | Non-program actions | |
| --- | --- | --- | --- | --- | --- |
| Approach | Scenarios | Ongoing | Planned | Ongoing | Planned |
| 1  Include all non-program actions. | Future with conservation (program + all other conservation actions | 1 | 1 | 1 | 1 |
|  | Future without program (stop program today, all other conservation actions continue | 0 | 0 | 1 | 1 |
| 2  Exclude all non-program actions | Future with only program (no other conservation actions) | 1 | 1 | 0 | 0 |
|  | Future without conservation | 0 | 0 | 0 | 0 |
| 3 | Future with program + non-program ongoing conservation actions only (no non-program planned actions) | 1 | 1 | 1 | 0 |
| Include only ongoing non-program actions | Future with ongoing non-program actions only (no program) | 0 | 0 | 1 | 0 |

*^a^* Numbers indicate whether a component of conservation (ongoing or planned program conservation actions vs. ongoing and planned non-program conservation actions) is included (1) or excluded (0) in the scenario. To calculate projected program impact, the bottom scenario is subtracted from the top scenario. For all 3 approaches, the components of conservation that contribute to this difference are only the ongoing and planned effects of the focal program, thus all approaches calculate the impact of the program on species recovery.

**
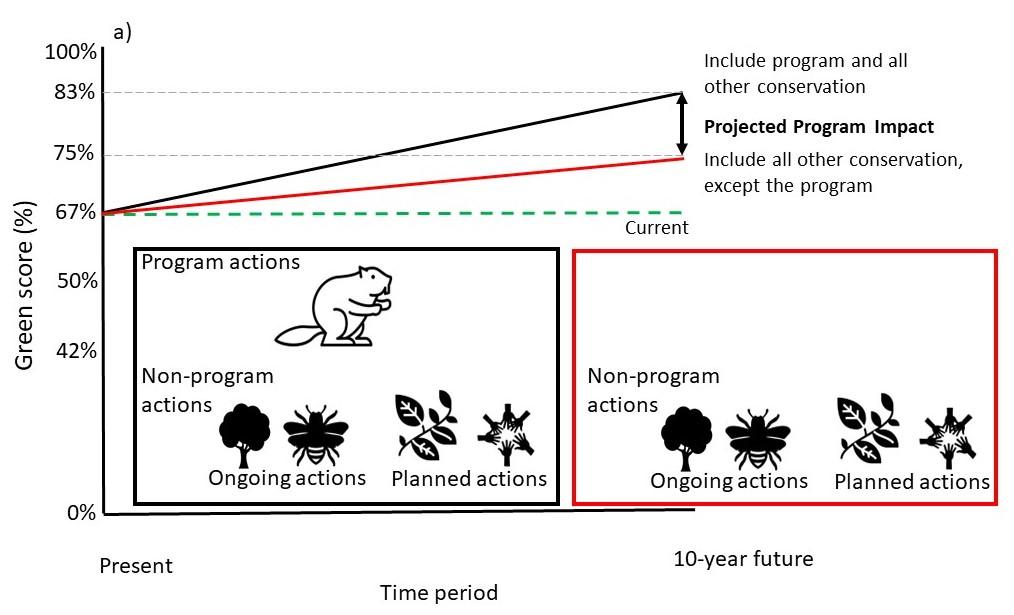

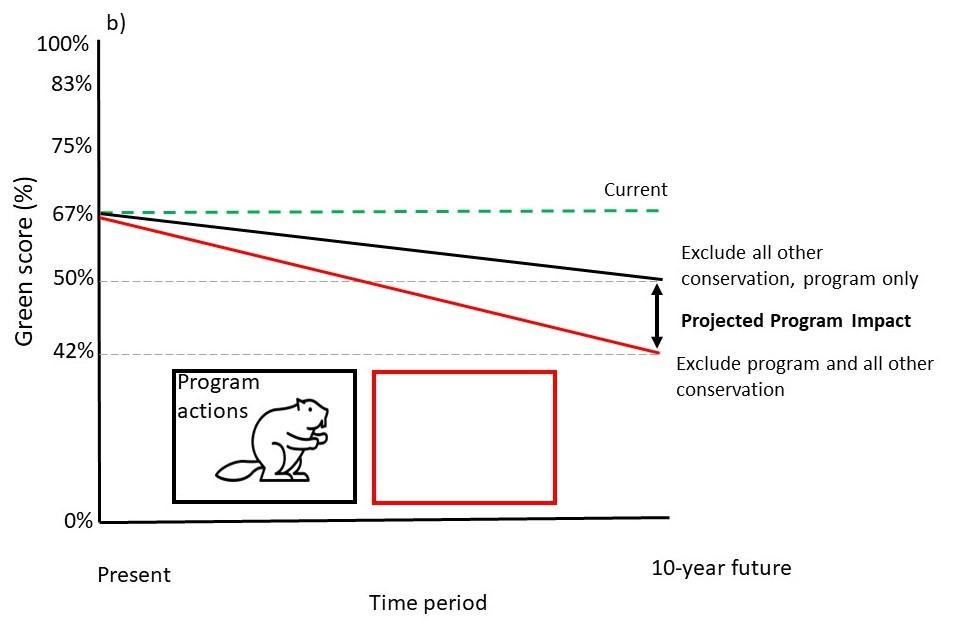

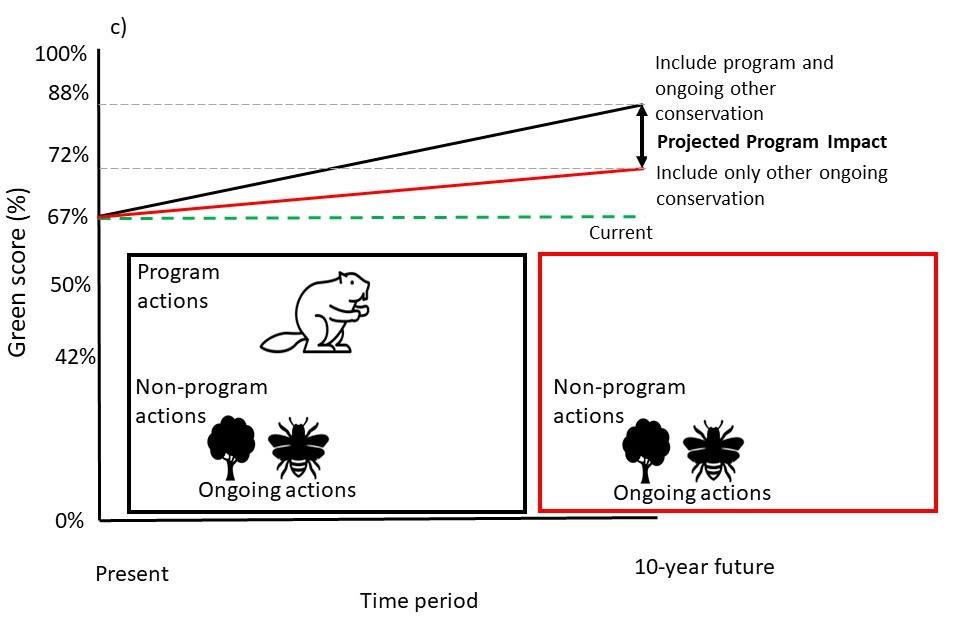
**

Figure S2: Visualisation of the three proposed methods for calculating projected program impact. Icons represent the focal program conservation actions (beaver reintroduction), ongoing non-program conservation actions (protected areas, planting for pollinators) and planned non-program conservation actions (invasive species removal and community engagement). a) Include all non-program actions: Include all non-program conservation actions in both short-term future scenarios, b) Exclude all non-program actions: Exclude all non-program conservation actions from both short-term future scenarios, c) Include only ongoing non-program actions: For both short-term future scenarios, include ongoing non-program conservation actions and exclude planned non-program conservation actions.

Table S2: Results of the conservation impact metrics for the global of global and program Green Status of Species preliminary assessments for the case studies tested in this manuscript. *^a^*

|  | Past |  | Short-term future *^b^* |  |  |  | Long-term future |  |
| --- | --- | --- | --- | --- | --- | --- | --- | --- |
| Species recovery program | Conservation Legacy (Global) | Program Legacy | Conservation Impact (Global) | Projected Program Impact Approach 1 (include all non-program actions) | Projected Program Impact Approach 2 (exclude all non-program actions) | Projected Program Impact Approach 3 (exclude ongoing non-program actions) | Long-term Aspiration | Potential Program Contribution to Long-term Ambition *^c^* |
| Telfair's skink (Island restoration program) | 17% | 17% | 14% | 5% | 5% | 5% |  |  |
| California condor (Baja California reintroduction program) | 20% | 7% | 7% | 0% | 0% | 0% | 53% | 7% |
| California condor (Breeding program) | 20% | 20% | 7% | 7% | 0% | 7% | 53% | 33% |
| Bolson tortoise (Breeding program) | 0% | 0% | 33% | 0% | 0% | 0% | 75% | 42% |
| Bolson tortoise (Biosphere reserve program) | 0% | 0% | 33% | 0% | 33% | 0% | 75% | 17% |
| Large antlered muntjac (Annamite mountains program) | 8% | 0% | 12% | 0% | 0% | 0% | 80% | 80% |
| Torrey pine (Torrey pine state park reserve program) | 28% | 0% | 0% | 0% |  |  | 73% | 20% |
| Annamite striped rabbit (Annamite mountains program) | 11% | 0% | 11% | 0% |  |  |  |  |
| Scimitar horned oryx (Reintroduction program) *^d^* | 4% | 4% |  |  |  |  |  |  |
| Hainan gibbon (Capacity building program) | 15% | 0% | 15% | 0% |  |  | 25% | 10% |
| Mountain yellow-legged frog (Northern population program) | 13% | 0% | 13% | 0% |  |  | 67% | 7% |
| Mountain yellow-legged frog (Southern population program) | 13% | 13% | 13% | 13% |  |  | 67% | 53% |
| Madagascan big-headed turtle (Rere restoration program) | 14% | 8% | 17% | 8% |  |  |  |  |
| Botsford's leaf litter frog (Vietnam frog conservation program) | 0% | 0% | 44% | 22% |  |  |  |  |
| Sterling's toothed toad (Vietnam frog conservation program) | 0% | 0% | 44% | 22% |  |  |  |  |
| Burmese star tortoise (Reintroduction program) | 8% | 8% | 28% | 20% |  |  |  |  |

*^a^* Results are preliminary as they have not undergone the full IUCN review process (global assessments), but all assessments were facilitated by a member of the Green Status of Species-SSC Integration Task Force, to ensure consistent application of the Green Status methodology.

*^b^* Initial testing included comparing the results of using approaches 1, 2 and 3 (include all non-program actions, exclude all non-program actions and include ongoing non-program actions from scenarios, respectively) to assess the future impact of conservation programs, as presented in rows 1-10. After approach 2 was deemed not a suitable method for assessing program impact, due to violation of a key assumption of independence of program and non-program conservation activities, and it was determined that approaches 1 and 3 produced consistent results, approach 2 was not used in subsequent testing, and assessors were given the option of using approach 1 or 3. All assessors used approach 1.

*^c^* Assessment of program contribution to long-term aspiration was considered in preliminary testing (Appendix 1), and results are presented for 9 species.

*^d^* As it is not a requirement for assessors to complete all scenarios in an assessment, there is no conservation impact of projected program impact for the scimitar-horned oryx assessment, as assessors chose to adopt this tool to assess the past impact of this program only.

## Appendix S3: Assessing impact over the longer-term

Attempting to assess program impact over 100 years is not typically appropriate due to the large number of assumptions which would be required to make estimates 100 years into the future of a program. The assumptions required to assess the potential future impact of a program over a 10-year period are likely to be more robust, and so, in most cases, this temporal scale should be used.

However, there are cases where it would often make it impossible to detect any program impact within 10 years, for example species with long generation lengths, or an age of first reproduction greater than 10 years. Certain interventions are also less likely to deliver measurable improvements in program GSS metrics within 10 years. For example, the time it takes for reintroduced individuals to be considered wild under Red List criteria and therefore to be included in a GSS assessment, could be longer than 10 years. It will also likely be harder to detect change within 10 years for newer programs, where infrastructure and systems need to be developed before interventions can begin. If a practitioner is unable to demonstrate any impact of planned work, they are unlikely to adopt this approach for impact evaluation.

We therefore considered how the impacts of a program could be assessed over a longer timeframe, where deemed necessary by assessors. We propose that, just as within the 10-year timeframe, the new scenario considered within a program evaluation is ‘Future without program (100 years)’. Unlike in the 100-year scenario in a global GSS assessment, which considers all possible conservation actions, for a program level assessment, this 100-year scenario would consider the expected state of the species 100 years into the future without the program as it stands today. This minimises the assumptions of the future state of the program, but allows assessors to evaluate its potential long-term impact. Case studies evaluating programs with the focal species with a generation length exceeding 15 years (Bolson tortoise (*Gopherus flavomarginatus*), Torrey pine (*Pinus torreyana*), Hainan gibbon and California condor) all failed to demonstrate any program impact within the 10-year time frame, but change was detected in 100 years (Table S2).

The approach differs from the global 100 years scenario, which considers the maximum plausible state of the species, constrained only by biological and socioeconomic restrictions. When considering the long-term future without the program, we assessed a scenario of what the state of the species in each spatial unit would be in 100 years’ time if the program, in its current state, were to stop. By assessing just the impact of the program in its current state, we aimed to mitigate the need for major assumptions about the future funding and possible interventions of the program, while allowing assessors to understand the potential impact of the actions within the program in the long-term.

Whilst we acknowledge that results of this 100-year time frame will be less robust than the shorter 10-year time frame, it can give an indication of what the program could achieve long-term. Results of looking at the long-term future have a different purpose: we would not advise using them to communicate what is expected from the program, but they have value in helping programs think about long term plans and measure ambition. There will still be instances where even in 100 years, no impact of the program can be determined, for example, if a program works within a very small proportion of the species range. Findings such as these are not necessarily negative, and could be used to incentivise conservation organisations to develop their programs to reach higher levels of impact. This may be achieved through by expanding the geographical scope of a program, changing or including more conservation actions within the program, or increasing collaboration to facilitate interventions with a greater impact.
